# CIN III lesions and regression: retrospective analysis of 635 cases

**DOI:** 10.1186/s12879-015-1277-1

**Published:** 2015-11-21

**Authors:** Melodi Motamedi, Gerd Böhmer, Heinrich H. Neumann, Reinhard von Wasielewski

**Affiliations:** Clinic of Plastic and Reconstructive Surgery, Sana-Klinikum Hameln, Saint-Maur-Platz 1, Hameln, D-31785 Germany; Institute of Cytology and Dysplasia (IZD) Hannover, Theaterstraße 14, Hannover, 30156 Germany; Gemeinschaftspraxis für Pathologie, Frankenburgstraße 31, Rheine, 48431 Germany; Institute of Pathology, Nordstadtkrankenhaus Hannover, Haltenhoffstraße 41, Hannover, 30419 Germany

**Keywords:** Cervical intraepithelial lesion, CIN III, Regression, HPV16

## Abstract

**Background:**

The rate of spontaneous regression in CIN III lesions is controversial. Whereas some studies have reported high regression rates of up to 38 % after prolonged biopsy-conus intervals, others have shown rates between 0 and 4 % without considering time intervals. Identification of young patients with potentially regressing CIN III could offer the chance to avoid conisation, thus lowering the risk of preterm labour.

**Methods:**

To further clarify the facts, we retrospectively compared 635 biopsies showing CIN III with the diagnosis of the conisation. Either regression (CIN I or less) or non-regression (CIN II and higher) was recorded. Diagnoses were made by light microscopy and p16 immunostaining.

**Results:**

Conisation was performed between 2 and 463 days after biopsy (median 8.9 weeks). Six hundred twenty one (98 %) were HPV-HR positive. In 345 cases, HPV subtyping was available, showing HPV16 infection in 57 %. Routine processing of the conisation tissue showed no corresponding CIN lesion (< CIN II) in 40 cases (6.3 %). Additional step sectioning of the tissue revealed small CIN II+ lesions in 80 %. Finally, eight cases (1.3 %) fulfilled the criteria of regression. No regression was seen in HPV16 positive cases. Twelve invasive carcinomas were detected by routine processing of the conisation tissue.

**Conclusion:**

These results are in contrast with some prior reports that might have overestimated spontaneous regression of CIN III. Study size and an accurate discrimination between CIN II and CIN III lesions by histopathology seem to be the most likely factors to explain the diverging results published. Complete step sectioning of the whole tissue is also mandatory in questionable cases. Although theories exist that the initial biopsy might stimulate the immune system, thus triggering regression within weeks, our data do not substantially support such a mechanism. Overall, the chance of a CIN III lesion to regress rapidly within weeks or months after diagnosis seems to be small. We found more previously undetected invasive cancer than we observed regression. Therefore, a change in the current policy to treat CIN III lesions is unwarranted.

## Background

Cervical cancer, one of the most frequent cancers affecting women, is in most cases triggered by cervical infection with human papilloma virus (HPV) high risk subtypes [1–4]. HPV-induced squamous intraepithelial lesions are regarded as precursor lesions and graded into three different risk groups (cervical intraepithelial lesion Grade I-III; CIN I-III). However, the natural history of the highest risk group (CIN III) has been controversially discussed over the last few decades [5]. It has been reported that less than half of CIN III will progress to invasive cancer [6]. Conisation is widely accepted as the therapy of choice for CIN III. This procedure is appropriate for lesions that would have persisted or even progressed to cancer [7]. However, there is little data available on the spontaneous regression of CIN III lesions [8–10]. Accurate identification of such spontaneously regressing CIN III lesions will have clear clinical benefits, as it would abrogate the need for patients to undergo conisation. Moreover, well known sequelae, such as elevated risk of preterm delivery could be reduced, which is important for young women not yet having concluded their family planning phase [11–14].

According to the published literature, the rate of natural regression in histologically approved CIN III lesions is difficult to estimate as therapeutic excision by cone biopsy eliminates all dysplastic tissue in most cases. It has been argued that careful monitoring without intervention would be unethical for CIN III lesions, as it holds the risk of the lesion developing into invasive cancer. In this context it is important to consider that the majority of women with CIN III will not develop cancer, raising the concern that >50 % of patients are over-treated at the moment [6].

It is therefore critical to develop procedures to better define CIN III lesions and thus lower the rate of over-treated women. In the recent literature, at least two groups have reported that, across all subtypes of HPV, approximately one third of CIN III lesions undergo complete regression in a timeframe of < 6 months after biopsy [8, 9]. Munk et al. reported that regression was more frequent if the interval between biopsy and conisation exceeded >8 weeks (2007). Whereas in the groups with short (0–4 weeks) and medium intervals (4–8 weeks) regression rates were shown to be 5 %, conisation specimens showed only CIN I lesions or less in 38 % of the long interval observation group (>8 weeks interval). The authors concluded that a prolonged interval could reduce the rate of beneficial conisation. In another paper from the same group investigating larger numbers of cases, the regression rate was suggested to be 18 % [15]. Even this lower percentage seems to be a substantial result that could justify new algorithms of CIN treatment. Trimble et al. included CIN II and CIN III lesions in their study and found a spontaneous histologic regression as high as 28 % after an observation period of 15 weeks, whilst observing no difference in regression rates between the CIN II and the CIN III lesions in their patients [16]. In contrast, more recent studies did not find a significant regression rate among high risk CIN lesions [10, 17].

This discrepancy warrants further investigation, especially when taking into account that most studies are dealing with a relatively small number of patients or have been published in the pre-p16 area. Thus, a few cases without immunohistochemical confirmation could have influenced the reported percentages of regression quite considerably. Another interesting aspect relates to the observation that regression rates depended on the time interval between diagnostic biopsy and therapeutic conisation [8]. Particularly this latter study prompted us to re-evaluate the regression rates of histologically approved CIN III lesions in a large cohort. The question posed was whether we could confirm the rate of time dependent regression rates in CIN III lesions, thus providing strong evidence to change our guidelines for the routine conisation programme.

## Methods

All patients initially showed PAP IIID (Munich II classification, corresponds to LSIL and HSIL of the Bethesda classification) or higher smears and were referred to our interdisciplinary centre for dysplasia. Cytology was performed either at our institution or locally. All further treatment (differential colposcopy, biopsy, conisation, histopathology) was carried out at our clinic. Referral criteria were in line with the national guidelines.

**Fig. 1:**
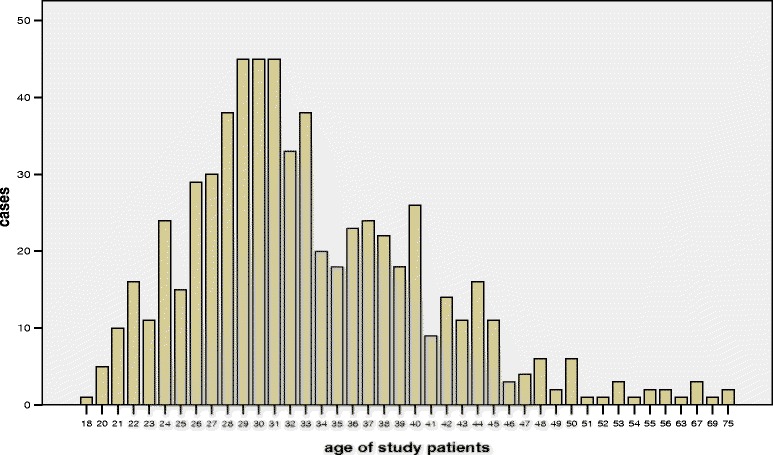
Age distribution of the 635 consecutive cases investigated

**Fig. 2:**
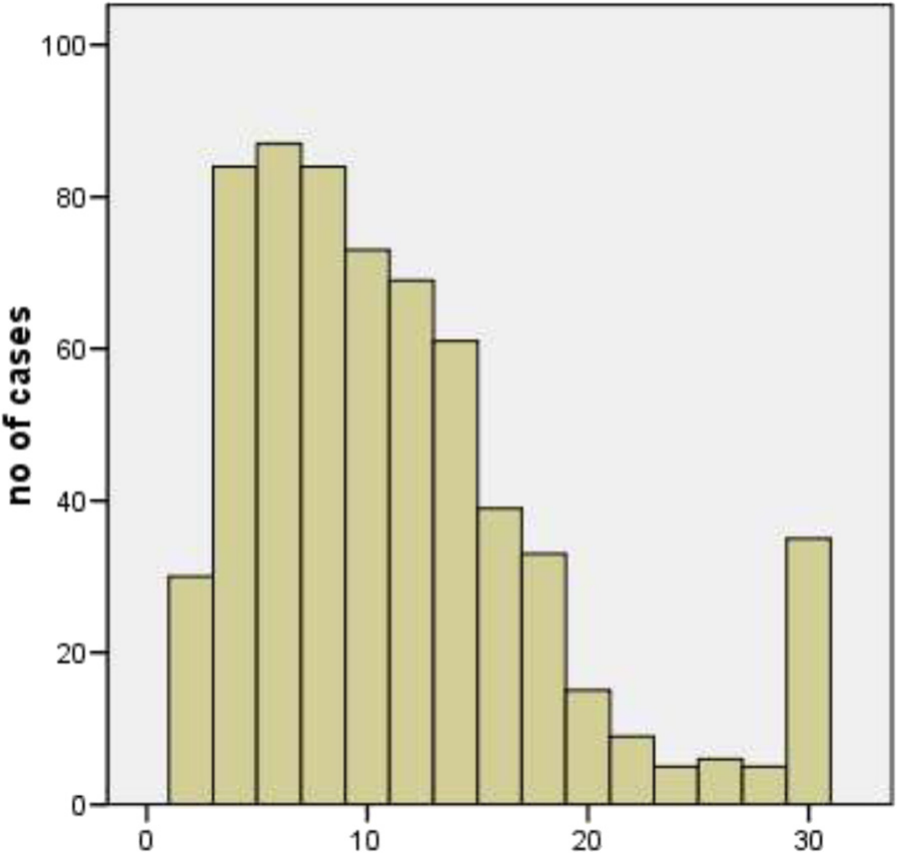
Interval between diagnostic biopsy and cone biopsy (*n* = 635). Intervals longer than 30 weeks are summed up in the last column. X-axis: weeks

The present study population consists of 635 consecutive women fulfilling the inclusion criteria as follows: age 18–75 years, biopsy-proven CIN III histology followed by conisation, diagnoses between 1 January 2008 and 30 April 2011, original diagnostic slides and paraffin blocks available. Most cases presented here were treated by LEEP (Loop Electrical Excision Procedure), some by laser conisation if necessary. No case was treated by cold-knife excision. After recruitment of cases, data were analysed in anonymised form. The Ethics committee of the Medical Association of Lower Saxony (Ärztekammer Niedersachsen) has declared that the present study is unaffected by the Helsinki Declaration (http://www.wma.net/en/30publications/10policies/b3/index.html). The Ethics Committee of the Medical School Hannover (Hannover, Germany) confirmed that there are no ethical or legal objections. Age, date of biopsy, size of biopsy, date of conisation and size of conisation were recorded and biopsy-cone interval was calculated (Figs. 1 and 2). In addition, HPV status (high risk subtypes, HR) was known for 621 cases (97.8 %) and in 2.2 % HPV status was unknown.

HPV testing was done either by Hybrid Capture 2 (HC2, Digene-Qiagen, Hilden, Germany; *n* = 276 cases) or by molecular testing of HPV DNA using the Roche human papillomavirus genotyping test on the Cobas 4800 (Cobas™; Roche, Mannheim, Germany; *n* = 345 cases) [18, 19]. All tests were performed according to the manufacturer’s instructions. The latter test is based on a real-time PCR using the primers GP5+ / GP6+ combined with hybridisation. It is an FDA approved and CE-marked test that detects 14 high-risk HPV DNA: HPV-16 and HPV-18 individually and the other 12 types pooled (31, 33, 35, 39, 45, 51, 52, 56, 58, 59, 66 and 68). Cells for molecular testing were collected on a cytobrush.

For better comparability with other studies, biopsy-cone intervals were divided into three or two groups. First, we used the 33rd percentiles of the observation intervals in the present study (study intervals = “SI”), thus dividing the patients into three groups of roughly the same size (SI: < 6.1 weeks, 6.1–12.0; >12 weeks). Secondly, we chose identical time frames to those published before by Munk et al. to enable direct comparison (Munk Intervals = “MI”: <5.4 weeks, 5.4–8.9 weeks and longer than 8.9 weeks [8]). Thirdly, we used a 15-week interval to distinguish two groups to facilitate comparison with the data of Trimble et al. [9, 16].

Diagnoses of the biopsy (CIN III) and the subsequent cone biopsy were compared. Histopathological diagnoses were made using the criteria published by the WHO in 2003 [20]. CIN III was diagnosed only if at least one mitotic figure could be proven in the upper third of the dysplastic epithelium, fulfilling the WHO criterion “Mitotic figures may be numerous and are found at all levels of the epithelium.” The recent edition of the WHO Blue Book was published after data collection of our study had been completed and the binary classification system of CIN (putting CIN II and CIN III together) suggested therein was therefore not applied [21]. Biopsies were taken in areas of abnormal pattern at punctum maximum. One, two, three or four biopsies were taken in 93, 6, 0.6 or 0.2 % of cases respectively. Biopsy size was on average 5.5 mm. Tissues were fixed immediately in neutral buffered formaldehyde for 16–36 h and routinely processed. Paraffin blocks were cut and 4 μm sections were stained with haematoxylin and eosin (HE).

All cases included in this study showed medium to strong p16 positivity in the diagnostic biopsy. P16 immunohistochemistry was performed and evaluated using CINTec© histology kit (clone E6H4; mtm Laboratories, Heidelberg, Germany) according to the manufacturer’s protocol using an automated staining system. Negative controls (normal cervix) and positive controls (CIN III) were included in each series. Staining was scored either negative or positive based on nuclear or cytoplasmic signals according to the LAST criteria [22, 23].

LEEP and laser conisations were fixed immediately as described above. After fixation, the cone was divided into twelve topographically designated sections (“clockface”), processed and always completely embedded in paraffin. Sections of all twelve segments were reviewed for diagnosis. Diagnoses of CIN II, III or higher were regarded as persistence or progression (non-regression cases). A diagnosis of CIN I or less (e.g. condyloma, inflammation) was regarded as regression. In all cases showing putative regression, the paraffin blocks of the cones were completely sectioned until no further tissue was left in the paraffin blocks. P16 staining was performed in areas of interest as described above. Overall, complete sectioning was necessary in 40 cases (6.3 %). In 42 cases, additional p16 stains were performed on conisation tissue.

**Fig. 3:**
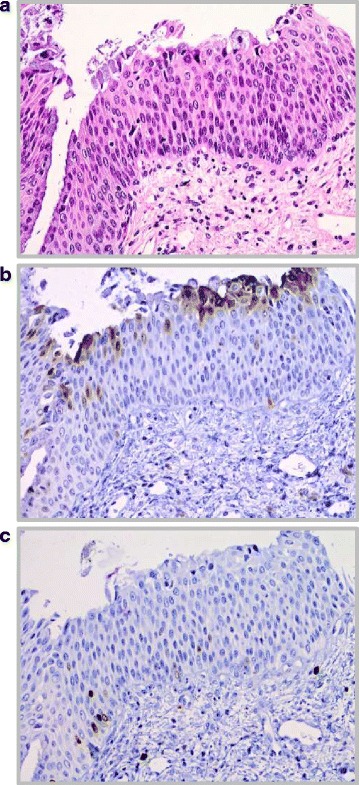
**a** H&E stain of atypical epithelium, showing hyperchromatic nuclei and transformed nucleus-cytoplasm ratio. Most of the squamous epithelium is covered by endocervical secretory cells, fulfilling the diagnosis of metaplasia. Intraepithelial lymphocytes mimic mitoses. **b** p16 immunohistochemistry. Same area as shown in Fig. 3a, the squamous epithelium is p16 negative, thus indicating that this lesion is not related to an HPV high-risk infection. **c** Ki-67 immunohistochemistry. Same area as shown in Fig. 3a and b. Only very few basal and parabasal cells are marked dark brown (physiological area). Intraepithelial lymphocytes in the upper half are negative

Cases were independently reviewed on a light microscope by two pathologists experienced in gynaecopathology (H.N., R.v.W.). Equivocal cases were decided on a multi-head microscope using additional immunohistochemistry if necessary (e.g. Ki-67, data not shown). In two cases showing equivocal changes of squamous epithelium, the p16 staining was negative. Additional Ki-67 staining showed a physiological pattern with positivity of a few cells in the proliferation zone. Thus, the lesions were diagnosed as atypical metaplasia (e.g. Fig. 3).

All patients with the diagnosis CINIII were offered and advised to undergo a prompt intervention. The prolonged time intervals between diagnostic biopsy and therapeutic conisation reported here are based solely on the patient’s choice (personal reasons). All patients were members of health insurance programmes, thus excluding any financial reason for the different time intervals.

## Results

**Fig. 4:**
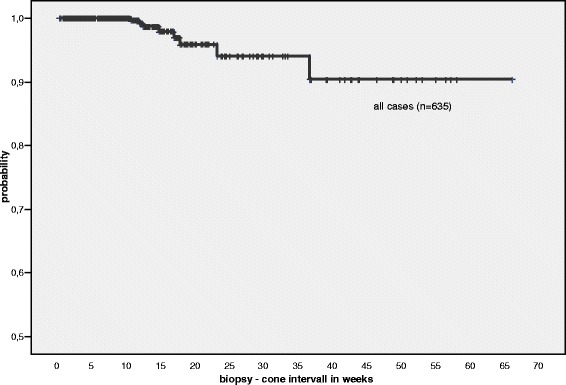
Kaplan-Meier plot showing the biopsy—cone intervals on the x-axis for all cases (weeks). The shortest interval for a case showing regression was 10.71 weeks, the longest interval was 36.71 weeks. Note that the y-axis ranges from 0.5 to 1.0

Among 1495 patients screened for this study, 635 patients fulfilled the inclusion criteria and had both a cervical biopsy showing a CIN III lesion (p16 positive) and a consecutive conisation at our clinic. The median age of our patients was 32 years (detailed age distribution is shown in Fig. 1). 97.8 % of cases were positive for HPV-HR. Among 345 patients with data for the HPV subtype, 197 were positive for HPV-16 (57 %) and 148 were negative for HPV-16 but HPV-HR positive (43 %; subtype 18 or “others”). Biopsy-cone interval for all qualified cases ranged from 2–463 days (0.2–66.1 weeks), the median interval was 62 days (8.9 weeks). In eight out of 635 cases CIN I or less was diagnosed in the follow-up cone (1.3 %), qualifying them as “regressed” (Fig. 4). CIN II was found in 39 cases (6.1 %), classified as “persistence”. Five hundred eighty eight cases (92.6 %) showed either identical CIN III lesion (persistence) or carcinoma (12/635, i.e. 1.9 %; persistence or progression). Table 1 summarises the data including the size of the biopsies, time intervals and age of the patients in the different subgroups.

**Table 1:** 635 cases were investigated. 627 showed no regression, i.e. persistence of CIN III (*n* = 576; 90.7 %), persistence of CIN II (*n* = 39; 6.1 %) or invasive carcinoma (progression or more likely missed by the previous biopsy: *n* = 12; 1.9 %). Eight cases showed regression according to our definition, six of them with the diagnosis of CIN I (0.94 %), one showing koilocytosis of the tissue without dysplasia (0.16 %) and one showing inflammation only (0.16 %). Mean size of biopsy was slightly smaller in the eight regression cases and slightly larger in the carcinomas. The few cases with regression and carcinoma had larger sizes of cone biopsies and the patients were on average older. The small numbers of the latter two subgroups result in a reduced significance for analytical purposes

	Size of biopsy (max)	Diameter cone (max)	Thickness cone (max)	Interval in weeks	Age
All cases / *n* = 635					
‐ mean	5.46	18.3	10.8	11.25	33.3
‐ SD	1.44	4.13	4.57	9.73	8.05
‐ median	5.0	18.0	10.0	8.86	32.0
Non-regression / *n* = 627					
‐ mean	5.46	18.31	10.81	11.17	33.25
‐ SD	1.44	4.14	4.58	9.72	8.07
‐ median	5.0	18.0	10.0	8.86	32.0
Regression / *n* = 8					
‐ mean	5.25	19.13	13.13	18.1	36.0
‐ SD	1.51	3.27	4.02	8.53	6.05
‐ median	5.0	19.5	12.0	15.93	38.0
Carcinomas / *n* = 12					
‐ mean	5,75	20.83	15.83	5.89	39.33
‐ SD	2.18	3.32	5.39	5.8	13.24
‐ median	5.0	21.5	15.5	3.85	36.0

A comparison of outcomes (“regression” or “non-regression”) correlated to the different time intervals described above is shown in Table 2a-c. Using our own study intervals, 2 out of the 8 patients with regression belonged to the intermediate group (6.1–12 weeks interval), the other 6 patients were in the longest interval group. Regression was not observed during the first 6.1 weeks (Table 2a). If intervals reported by Munk et al. [8] were used, regression was observed only in patients with 8.9 weeks or longer between punch biopsy and conisation (Table 2b). Trimble et al. investigated cases after a 15-week interval and we have divided our cases accordingly for adequate comparison (Table 2c).

**Table 2:** a,b,c Regression and non-regression in CIN III according to different time intervals

a	Biopsy—cone intervals using 33 % percentile of this study			
Follow-up	0–6.1 weeks	6.14–12 weeks	>12 weeks	Total
Non-Regression	215 (34.3 %)	210 (33.5 %)	202 (32.2 %)	627 (98.74 %)
Regression	0 (0 %)	2 (0.9 %)	6 (2.9 %)	8 (1.26 %)
Total	215 (33.8 %)	212 (33.4 %)	208 (32.8 %)	635 (100 %)
b	Biopsy—cone intervals according to Munk et. al. ([8])			
Follow-up	0–5.4 weeks	5.4–8.9 weeks	>8.9 weeks	Total
Non-Regression	180 (100 %)	141 (100 %)	306 (97.45 %)	627 (98.74 %)
Regression	0	0	8 (2.55 %)	8 (1.26 %)
Total	180 (28.35 %)	141 (22.2 %)	314 (49.44 %)	635 (100 %)
c	Biopsy—cone intervals according to Trimble et al. [9, 16]			
Follow-up	<15 weeks		≥15 weeks	Total
Non-Regression	504 (99.21 %)		123 (96.85 %)	627 (98.74 %)
Regression	4 (0.79 %)		4 (3.15 %)	8 (1.26 %)
Total	508 (80.0 %)		127 (20.0 %)	635 (100 %)

a) three groups each representing a 33 % percentile of the time intervals of this study. 6 of 8 regression cases were observed in the group with intervals longer than 12 weeks, representing 2.9 % in this subgroup. b) Time intervals were chosen exactly as published by Munk et. al. to enable a direct comparison. All regression cases occurred in the longest interval group. The regression rate in this subgroup was lower than in any of the intervals investigated in the study of Munk et al. c) An analysis of only those cases showing an interval of 15 weeks or longer makes it possible to compare the regression rates with the data of Trimble et. al. [9, 16]. Our study found 3.15 % of regression among 127 cases evaluated in this subgroup

**Fig. 5:**
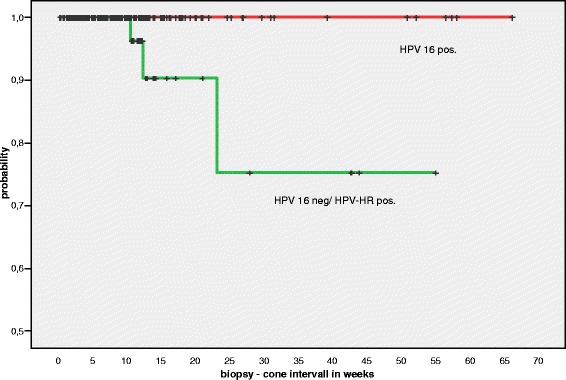
Subanalysis of those 345 cases tested by the Cobas 4800 system. Patients with HPV16 positive lesions never showed regression in our series. Note the scale of the y-axis (0.5 –1.0). X-axis: biopsy—cone interval in weeks

In the group of patients with HPV16 infection (*n* = 197), no regression occurred (0 %), whereas in the non-HPV16 group (HPV18 or others; *n* = 148) three cases showed regression (2 %; Fig. 5; Table 3). The five other regression cases belonged to the group of cases known to have an HPV-HR infection, but no information regarding the subtype was available (*n* = 276; 1.8 % with regression).

**Table 3:** Cone diagnoses in histologically proven CIN III lesions (biopsy) according to HPV subtype and biopsy-cone interval

	Biopsy-cone interval			
	<6.1 Weeks	6.14–12 Weeks	>12 Weeks	Total
HPV 16+				
≤ CIN I	0	0	0	0
CIN II	4 (5.8 %)	2 (3.1 %)	6 (9.4 %)	12 (6.1 %)
CIN III	65 (94.2 %)	62 (96.2 %)	58 (90.6 %)	185 (93.9 %)
Total	100	175	255	197
HPV-HR pos. but HPV16 neg				
≤ CIN I	0	1 (2.1 %)	2 (4.4 %)	3 (2.0 %)
CIN II	2 (3.6 %)	3 (6.3 %)	5 (11.1 %)	10 (6.8 %)
CIN III	53 (96.4 %)	44 (91.7 %)	38 (84.4 %)	135 (91.2 %)
Total	55	48	45	148

Subanalysis of only those 345 cases with known HPV subtype (16, 18, or other) according to biopsy-cone intervals. CIN I and less were rated as “regression” and CIN II/CIN III as “non-regression”

In twelve cases, invasive carcinoma was detected in the cone biopsies during routine processing not previously known. Table 4 summarises further histopathological data available for those cases.

**Table 4:** Twelve carcinomas previously unknown were detected in the cone biopsies

Carcinomas / HPV 16^a^	Biopsy size	pTNM, R-status, Grading	Interval in weeks	Age
‐ 1	5	pT1a1(m), L0,V0,R0; G2	6.14	47
‐ 2 / yes	7	pT1a1, L0,V0,R0; G2	3.43	35
‐ 3	7	pT1a1(m), L0,V0,R0; G2	2.0	37
‐ 4	5	pT1b1, L0,V0,R0; G3	12.0	32
‐ 5 / yes	5	pT1a2, L0,V0,R0; G2	1.43	26
‐ 6	9	pT1b1, L0,V0,R1; G3	3.57	32
‐ 7 / yes	8	pT1a1, L0,V0,R0; G2	6.71	29
‐ 8	1	pT1a1, L0,V0,R0; G2	1.71	49
‐ 9 / no	5	pT1a1, L0,V0,R0; G2	4.0	40
‐ 10 / yes	5	pT1a1(m), L0,V0,R0; G2	3.71	40
‐ 11 / no	8	pT1a2, L0,V0,R1; G2	4.0	75
‐ 12 / yes	4	pT1a1, L0,V0,R0; G2	22.0	30

The table shows the biopsy size of the initial biopsy (CIN III) in mm

^a^In the first column, HPV 16 status is listed for those seven cases investigated by the Cobas 4800 system

## Discussion

CIN III is regarded as a true pre-cancer, characterised by transformation to invasive cervical carcinoma at a rate of 0.2 to 4 % within twelve months [24]. Earlier estimations for the overall risk have been as high as 12 % (follow-up period between 1–20 years, no detailed data available) [25]. The latter paper is based on observations between 1956 and1989, the majority of them published before 1968. Given the common agreement to treat CIN III lesions by surgical removal of transformed tissue, validated knowledge about the natural history of these lesions is surprisingly limited. A single study has been published by McCredie et al., reporting the follow-up results of an unethical clinical study in New Zealand [6]. Women with histologically confirmed CIN III lesions by punch or wedge biopsies and with proof of persisting disease (cytology) were left untreated (*n* = 92). Fifty percent developed cancer within 30 years (31 % after 10 years). No data on persistence or regression were available. It seems reasonable and essential to treat a precursor lesion of cancer associated with a 30 % chance to develop cancer within the next 10 years.

On the other hand, however, Ostör has reported a 32 % regression rate of CIN III lesions [25]. Moreover, in recent years, regression rates between 26 and 38 % have been published [8, 9, 16]. Among the latter studies, especially the report of Munk et al. seems to be interesting, since the regression interval was time-dependent. The highest regression rate was observed in the subgroup of patients with biopsy-cone intervals longer than 9 weeks. It was speculated that the diagnostic biopsy might have a positive effect on the natural history of the CIN III lesion by revealing previously undetected viral antigens and thus triggering an effective immune response and subsequent lesion clearance [8]. This model aligns with their observations, leading to the conclusion that a longer biopsy-cone interval could lower the rate of non-beneficial conisation. Therefore, the authors conclude that a longer biopsy-cone interval should be accepted to obtain the highest regression rates. In the end, unnecessary cone-biopsy procedures could be prevented. However, the study by Munk et al. was hampered by the small number of cases investigated. Only 21 cases belonged to the group with a biopsy-cone interval of 8.9 weeks or longer, eight of which showed regression (38 %).

The present study was designed to retrospectively analyse regression rates in a much larger cohort of patients (*n* = 635). Among these, 314 cases belonged to the group with a biopsy-cone interval of 8.9 weeks or longer (Table 2). In contrast to the study of Munk et al., all biopsy diagnoses were ascertained by p16 immunohistochemistry. Moreover, all cone excisions showing putative regression by routine work-up were entirely sectioned until no further tissue was left. The latter procedure was performed in 40 cases (6.3 % overall) and discovered in 32/40 (80 %) of persistent CIN II/III lesions (i.e. “non-regressors”). Although Munk et al. gave a detailed description of the formal protocol to process cone excisions, no data were available about step sectioning [8]. Among the 635 consecutive CIN III cases investigated here, only eight cases showed proof of regression (1.3 %). Interestingly, we observed no single case of regression during the first 8.9 weeks, but the few regressions occurred after an interval of 9 weeks or longer. Seven of eight patients (87.5 %) were older than 30, although this age group represented only 57.6 % of all patients. Only one case of true regression was seen in the group of patients 30 years or younger, resulting in a regression rate of 0.4 % in this subgroup (1/259). In 345 cases, subtyping of HPV was available. Three cases in this group showed regression (3/345; 0.9 %). Among HPV16 positive cases, no regression was observed (0 %), compared to non-HPV16 cases (2 %). In the study of Munk and colleagues, age and HPV status were not mentioned [8].

Other studies have shown regression rates during the biopsy-cone interval of 0, 2.2 or 1.47 % [10, 26, 27], investigating 139, 45 and 339 cases respectively. These results are in good relation to our observations. However, including data of the biopsy-cone time intervals, the present study design allows a direct comparison to the data of Munk et al. [8].

What are the putative reasons to explain the differences between the studies? First, the small number of cases investigated by Munk et al. might have exaggerated the impact of single events that could potentially occur by chance. The reliability of a CIN III diagnosis in both the small biopsies and the cone is challenged by histological changes that might mimic CIN (e.g. metaplastic changes, inflammation with atypia) that can be excluded by the use of p16 immunohistochemistry [28, 29] as has been done in our study. Moreover, the differentiation between CIN II and CIN III lesions (both p16 positive) is sometimes difficult and not well defined. In our cases, we applied the criteria of the 2003 WHO Blue Book [20]. If emphasis was put on the feature of mitotic figures in the upper third of the epithelium to satisfy the diagnosis of CIN III in addition to cellular and architectural atypia, differentiation of CIN II and CIN III lesions were not difficult. For CIN II lesions, however, it is well documented that the rate of regression is much higher [27, 30, 31]. From a logical point of view, it does not seem to make much sense first to merge two grades with different risk profiles and then report about high regression rates as a new finding. Our data presented here suggest that strictly applying the known criteria identifies those patients with little chance of regressing rapidly. Expanding the cytological category of HSIL to histopathology may be unwarranted from a biological and therapeutic point of view [27, 29, 32–35], but has been done by many studies [9, 16, 28, 29]. It does not matter whether the regression rate of CIN III is 1.3 % or higher, to date there has been no convincing approach to identify the “regressors”. Our finding of more carcinomas by routine processing of cone tissue than regressed cases among our study population hints towards another dilemma: prolonging the biopsy-cone interval would delay appropriate treatment of so-far undetected invasive carcinomas.

## Conclusion

Rapid regression of p16-confirmed CIN III is a rare event within an interval of several months after histological diagnosis. This is especially true for younger patients (<30y) and was never observed in patients infected by HPV16. Previous studies showing much higher regression rates within 9–20 weeks might have overestimated the percentage rate, presumably due to small study size and methodological shortcomings. The regression cases observed in our study occurred after longer intervals, which might be explained by an immunological stimulation via wound healing after diagnostic biopsy procedure. Even if true, this was a rare effect in our study, that was much smaller than published previously. We cannot rule out, however, that a wait-and-see strategy could be effective at much longer intervals. However, a substantially prolonged interval would not solve the problem of identifying those (few) patients with true regression but pose new problems. So far undetected carcinomas would be left untreated and new carcinomas could develop [8, 10, 26, 27]. All in all, we do not see any evidence to change the current treatment strategy of biopsy-proven CIN III lesions.

## Abbreviations

CIN: Cervical intraepithelial neoplasia
MI: Munk interval
SI: Study interval
HSIL: High-grade intraepithelial lesion
